# The Efficacy of Prospective Memory and Metacognitive Skills Training in Improving the Cognitive Skills and the Quality of Life of Elderly Persons With Dementia

**DOI:** 10.7759/cureus.67454

**Published:** 2024-08-22

**Authors:** Punitha P, Shrishte AS

**Affiliations:** 1 Occupational Therapy, Saveetha College of Occupational Therapy, Saveetha Institute of Medical and Technical Sciences, Saveetha University, Chennai, IND

**Keywords:** qol, dementia, cognitive skills, metacognitive skills training, prospective memory

## Abstract

Introduction

Elderly people may experience a deterioration in cognitive function as part of natural aging, which impacts their ability to function independently. Dementia is often experienced by the elderly; their cognitive and memory deficits can limit independence and productivity. Metacognitive skills training can facilitate self-awareness and strategy use and may improve cognitive skills.

Aims and objectives

The article aimed to evaluate the effectiveness of prospective memory and metacognitive skills training in improving cognitive skills and quality of life for elderly persons with dementia.

Methods

This was a quasi-experimental study that took place in Chennai city, India. Based on the criteria, a total of fifty (n = 50) elderly participants were selected and divided into control (n = 25) and experimental (n = 25) groups. The control group underwent conventional occupational therapy, whereas the experimental group underwent prospective memory and metacognitive skills training (PM and MST) over 36 sessions (three times/week, for 12 weeks). Outcome measures used were the mini mental status examination (MMSE) and quality of life - Alzheimer's disease (QOL-AD). Data were analyzed using the Mann-Whitney U test and Wilcoxon signed-rank test.

Results

The results revealed that there were statistically significant (p-value < 0.05) differences between control and experimental groups. When compared to the control group, the experimental group had greater significant improvement in cognitive skills (MMSE, the control group's mean score was 20.94 and the experimental group's mean score was 30.94, p-value = 0.026), and quality of life (QOL-AD, the control group's mean score was 13.54 and the experimental group's mean score was 37.46, p-value = 0.000) after the implementation of a 12-week therapy program.

Conclusion

This study concludes that PM and MST can be used as an effective intervention as it improves cognitive skills and quality of life among elderly persons with dementia.

## Introduction

Aging is a physiologically inevitable process with chronological, social, and psychological dimensions [1]. Occupational therapists work with elderly people with dementia to assess their areas of needs and capabilities and then design individualized therapy programs with the goal of extending their level of independence in daily life and enhancing their quality of life (QOL) [2].

Dementia is not a specific disease but rather a general term for the impaired ability to remember, think, or make decisions that interfere with everyday activities. Though dementia mostly affects elderly people, it is not considered part of the normal aging process [3]. Dementia can be caused by a number of diseases as they gradually damage the brain and kill nerve cells, leading to a decline in cognitive function, thinking, memory, and reasoning that becomes so severe that it affects a person's day-to-day activities [4].

Persons with dementia experience cognitive impairments that are severe enough to be observed by them or by their family and friends and that greatly interfere with day-to-day activities [5]. Very low cognitive abilities are associated with a lower QOL, limited functional independence, and substantial economic costs [6]. In clinical, research, and community contexts, the mini mental status examination (MMSE) is a frequently used brief screening tool that provides an overall measure of cognitive impairment [7].

QOL is a term used to refer to an individual’s total well-being [8]. Research indicates that dementia affects the QOL of patients in different ways, based on the severity of the illness, the type of care they receive, as well as their personality before developing the disease [9]. The elderly with dementia may develop problems like agitation, depression, anxiety, disinhibition, and irritability, and the consequences of these difficulties are likely to impair quality of life [10]. The QOL-Alzheimer's disease (QOL-AD) is a brief, 13-item measure designed specifically to obtain a rating of the patient's QOL for individuals with dementia. This measure focuses on QOL domains thought to be important in cognitively impaired older adults [11].

A study done by Shum et al. (2011), found that prospective memory can be improved in patients with traumatic brain injury using a compensatory approach with a relatively short duration and low intensity of intervention [12]. Similarly, Pikouli et al. (2023) found that metacognitive strategy training can improve decision-making abilities in older adults with mild cognitive impairment [13]. By analyzing these articles, it was found that there was no study to combine prospective memory (PM) rehabilitation and metacognitive skills training (MST) for patients with dementia. Hence this study focused on implementing PM and MST to improve cognitive skills and QOL among elderly persons with dementia.

## Materials and methods

The study received ethical approval from the Institutional Scientific Review Board of Saveetha College of Occupational Therapy (SCOT/ISRB/016/2023) and was conducted in Aanandham Old Age Home, Chennai, India. A quasi-experimental study design was adopted and 50 participants were recruited from old-age homes through convenience sampling. 

Patient characteristics

Both male and female participants aged above 65 with cognitive deficits (MMSE score < 20) and poor QOL-AD were included. This age group was included since aging is well established to promote the deterioration of cognitive function [14]. Persons with uncontrolled cardiorespiratory or metabolic disease, communication difficulties, and subjects with visual and hearing impairments were excluded from the study.

Outcome measures

The outcome measures used were the MMSE and QOL-AD. The MMSE is a brief cognitive screening tool with a total score of 30, and QOL-AD is a brief scale to obtain a patient's QOL with a total score of 52. Both the scales have good reliability and validity. 

Procedure

Informed consent was obtained from the patients as well as from the concerned centers, and the procedure was explained to the patients. Fifty elderly patients with dementia who have mild to moderate cognitive impairment were selected and randomly allocated to the control group (n = 25) and the experimental group (n = 25). A pre-test of the control and experimental groups was taken using MMSE and QOL-AD to assess cognitive function and QOL. Both the control and experimental groups underwent 45-minute sessions for 12 weeks (three days a week). The control group underwent conventional occupational therapy intervention, while the experimental group engaged in PM and MST activities.

**Figure 1:**
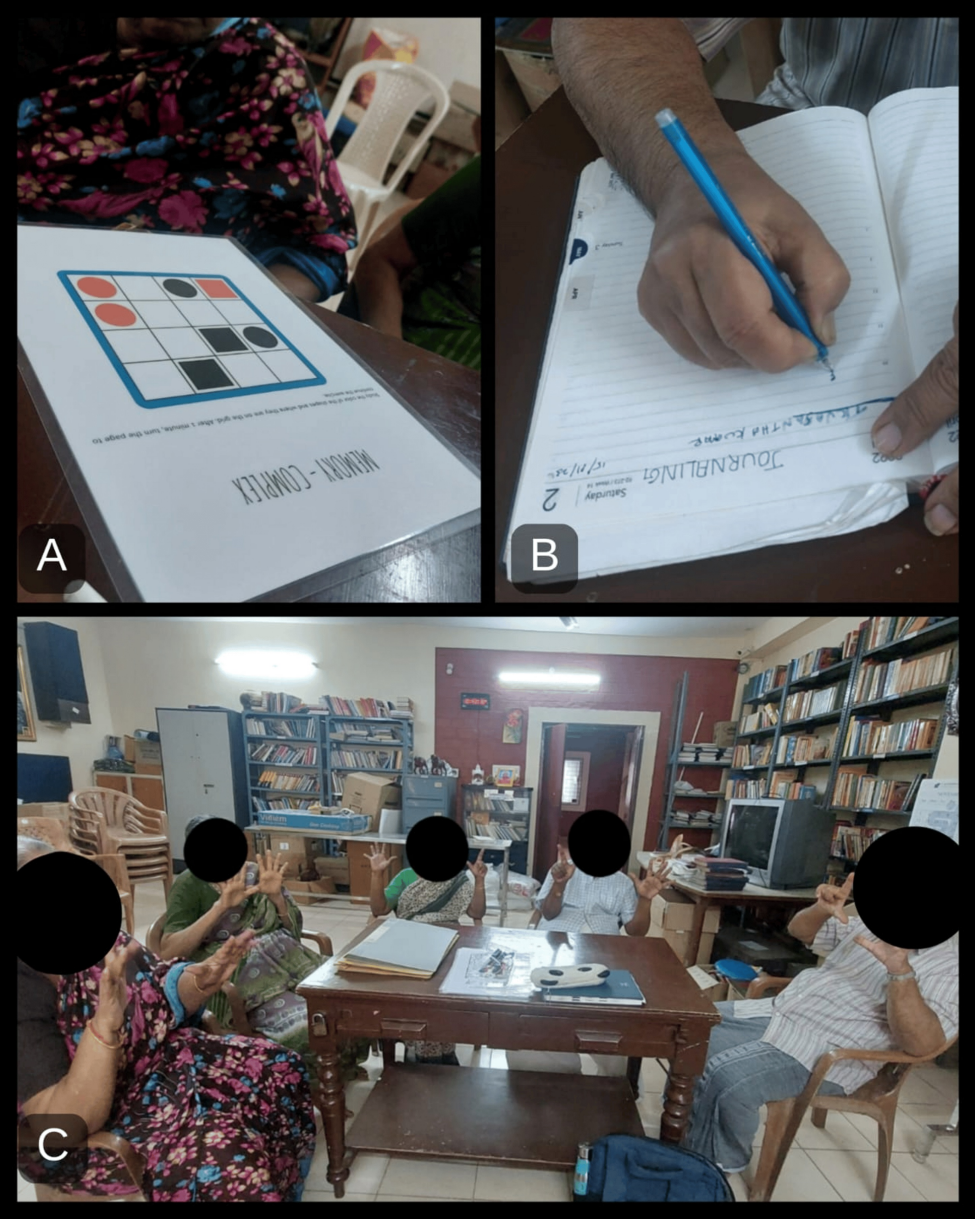
PM and MST activities A) Cognitive worksheets, B) journaling, and C) arithmetic calculations PM: prospective memory; MST: metacognitive skills training

**Figure 2:**
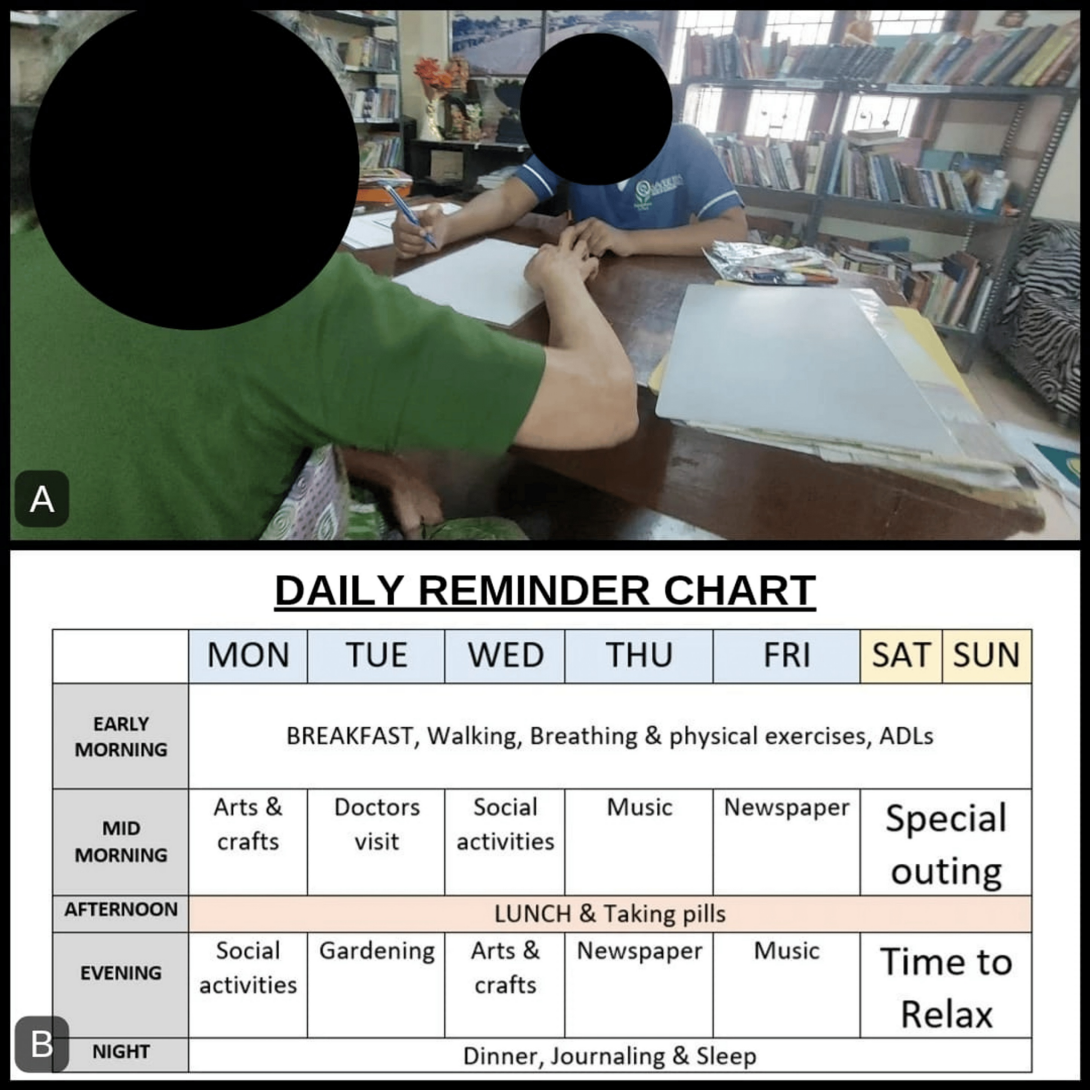
Compensatory techniques A) Education in compensatory strategies and B) daily reminder chart ADL: activities of daily living

In the experimental group, 45 minutes were split into five minutes of warm-up activities, 35 minutes of active phase activities, and five minutes of wind-down activities. The warm-up and wind-down activities included brain gym activities, shaking hands, clapping, etc., and the active phase included PM and MST activities such as cognitive workbooks (Figure 1A), journaling (Figure 1B), arithmetic calculations (Figure 1C), reading magazines, maze activities, role modeling, activity scheduling, self-monitoring, etc., followed by education in compensatory techniques like using memory aids, note-taking, setting alarms, etc. (Figure 2A). Daily reminder charts were also given (Figure 2B). After three months, post-tests for both the experimental and control groups were conducted using the same MMSE and QOL-AD scales. The statistical analysis was done with the help of IBM SPSS version 26.0 [15]. Since the sample size was 50 with convenience sampling, non-parametric methods like the Mann-Whitney U test and Wilcoxon signed-rank test were used to test the statistical differences between pre-test and post-test scores of Group A and Group B.

## Results

Since the p-value of 0.000 was less than 0.05, as given in Table 1, we accepted the alternate hypothesis. Therefore the statistically significant difference in the control group between pre-test and post-test scores of MMSE indicated the improvement in cognitive skills due to conventional occupational therapy program. 

**Table 1:** Difference between pre-test and post-test scores of the control group in MMSE *Significant at 5% (p < 0.05) level MMSE: mini mental status examination

Group	Test	Mean	N	Z value	p-value
MMSE Control group	Pre-test	14.2	25	-4.427	0.000*
	Post-test	16.48	25		

Since the p-value of 0.000 was less than 0.05, as given in Table 2, we accepted the alternate hypothesis. Therefore the statistically significant difference in the experimental group between pre-test and post-test of MMSE indicated the improvement in cognitive skills due to PM and MST. 

**Table 2:** Difference between pre-test and post-test scores of the experimental group in MMSE *Significant at 5% (p < 0.05) level MMSE: mini mental status examination

Group	Test	Mean	N	Z value	p-value
MMSE Experimental group	Pre-test	13.88	25	-4.425	0.000*
	Post-test	18.84	25		

Since the p-value of 0.026 was less than 0.05, as given in Table 3, we accepted the alternate hypothesis. Therefore the statistically significant difference in post-test scores of MMSE between the experimental and control groups indicated that there was a remarkable improvement in cognitive skills in the experimental group compared to the control group. 

**Table 3:** Difference between post-test scores of control and experimental group in MMSE *Significant at 5% (p < 0.05) level MMSE: mini mental status examination

Test	Group	Mean	N	U value	p-value
MMSE Post-test	Control	20.94	25	-2.223	0.026*
	Experimental	30.06	25		

Since the p-value of 0.000 was less than 0.05, as given in Table 4, we accepted the alternate hypothesis. Therefore the statistically significant difference in the control group between pre-test and post-test of QOL-AD indicated the improvement in quality of life due to conventional occupational therapy program. 

**Table 4:** Difference between pre-test and post-test scores of the control group in QOL-AD *Significant at 5% (p < 0.05) level QOL-AD: quality of life - Alzheimer's disease

Group	Test	Mean	N	Z value	p-value
QOL-AD Control group	Pre-test	23.12	25	-4.434	0.000*
	Post-test	27.16	25		

Since the p-value of 0.000 was less than 0.05, as given in Table 5, we accept the alternate hypothesis. Therefore the statistically significant difference in the experimental group between pre-test and post-test of QOL-AD indicated the improvement in QOL due to PM and MST.

**Table 5:** Difference between pre-test and post-test scores of the experimental group in QOL-AD *Significant at 5% (p < 0.05) level QOL-AD: quality of life - Alzheimer's disease

Group	Test	Mean	N	Z value	p-value
QOL-AD Experimental group	Pre-test	23.08	25	-4.385	0.000*
	Post-test	34.36	25		

Since the p-value of 0.000 was less than 0.05, as given in Table 6, we accepted the alternate hypothesis. Therefore the statistically significant difference in post-test scores of QOL-AD between the experimental and control groups indicated that there was a remarkable improvement in the QOL of the experimental group compared to the control group.

**Table 6:** Difference between post-test scores of the control and experimental group in QOL-AD *Significant at 5% (p < 0.05) level QOL-AD: quality of life - Alzheimer's disease

Test	Group	Mean	N	U value	p-value
QOL-AD post-test	Control	13.54	25	-5.822	0.000*
	Experimental	37.46	25		

## Discussion

Impairment of cognitive functions is common in elderly people with dementia, and it may hinder their QOL. This was supported by Han et al. (2022), who conducted a cross-sectional study on the risk factors that affect cognition in elderly people aged 65 and over [16].

The purpose of this study was to enhance cognitive ability and quality of life for elders with dementia using PM rehabilitation and MST. Outcome measures used in this study were selected based on the following studies done by Truong et al. (2024), who examined the validity of the MMSE and its domains using network analysis and supported the clinical usage of the MMSE [17]. Similarly, Thorgrimsen et al. (2003) conducted a study to assess the reliability and validity of the QOL-AD scale and concluded that the QOL-AD has very good psychometric properties and can be administered for people with a wide range of severity in dementia [18]. This study was done with 50 elderly dementia patients based on the study of Grigoria et al. (2023), who conducted a six-month follow-up randomized controlled trial where 45 participants were randomly allocated into two groups: the metacognitive training program and the cognitive exercise program, and further concluded that the positive effects of the metacognitive training program were evident over six months [19].

Tables 1, 4 show the difference between the pre-test and post-test scores of the control group and suggest that the intervention received by the control group showed improvement in post-test scores. The positive results are in accordance with a previous study done by Lim et al. (2012) to determine the benefits of a cognitive training program in improving the functional abilities of elders with mild cognitive deficits and provided some evidence for teaching strategies for mildly impaired seniors using cognitive stimulation and memory encoding techniques within the context of everyday tasks [20]. Dooley et al. (2004), in another study, concluded that Individualized occupational therapy intervention based on the person-environment fit model appears effective in improving the QOL of persons with Alzheimer’s disease [21]. Similarly, Kumar et al. (2014) concluded that a novel occupational therapy program improved the QOL of older patients with mild to moderate dementia in the physical and psychological components of the World Health Organization Quality of Life Brief Version (WHOQOL-BREF) scale [22].

Tables 2, 5 show the difference between the pre-test and post-test scores of the experimental group and suggest that the intervention received by the experimental group showed significant improvement in the post-test. Previous research by Rosi et al. (2019), got the same results concluding that metacognitive-strategy training helps to improve the decision-making skills of older adults with mild cognitive impairment and dementia [23].

Table 3 shows the comparison of post-test scores of MMSE between the control and experimental groups and suggests that there was a statistically significant improvement in the experimental group. The positive findings are supported by Rice et al. (2022), who conducted a systematic review of PM interventions across stages of dementia-related disorders by examining 21 studies on the success of intervention strategies for PM in patients with dementia. Although all the studies showed that therapies had positive effects on PM abilities, there are still concerns about the interventions' ecological validity, how long the benefits last, and how diverse they are for different stages of dementia-related diseases [24]. Mendrofa et al. (2020) conducted a study on the effectiveness of brain gyms in improving cognitive function in dementia patients. The cognitive function before and after brain exercises differed significantly (p-value < 0.05), and the findings indicated that brain exercise has an impact on enhancing cognitive function in older adults with dementia [3]. 

Table 6 shows the comparison of post-test scores of QOL-AD between the control and experimental groups and suggests that there was a statistically significant improvement in the experimental group. The positive findings are in accordance with Fleming et al. (2022), who conducted a study to find out whether compensatory training plus MST (COMP-MST) is effective in enhancing and preserving daily PM performance and the degree of psychosocial integration in persons with moderate-to-severe traumatic brain injury. Participants in all three groups saw improvements in primary outcomes that were clinically relevant [25].

From the results, it has been observed that intervention and the daily practice of PM and MST activities result in a noticeable improvement in the cognition of elders with dementia and enhances their QOL simultaneously. So, PM and MST training can be applied by using a variety of tasks to enhance cognition and QOL.

However, this study has some limitations: it was done with a small sample size, and the result could not be generalized as we employed convenient sampling. Future researchers should focus on more diverse samples and a large sample size to replicate and extend our findings.

## Conclusions

Elderly persons with dementia are more likely to demonstrate poor QOL, which may be a consequence of deficits in attention, orientation, memory, and various other cognitive impairments. They can regulate themselves positively only if they know the metacognitive strategies. The results obtained from this study add evidence to the effectiveness of PM and MST in improving cognitive skills and QOL of patients with dementia. Also, PM and MST can be incorporated into occupational therapy programs to improve cognitive skills and QOL among elderly persons with dementia.
